# Effect of Gut Microbiota-Directed Complementary Food Supplementation on Fecal and Plasma Biomarkers of Gut Health and Environmental Enteric Dysfunction in Slum-Dwelling Children with Moderate Acute Malnutrition

**DOI:** 10.3390/children11010069

**Published:** 2024-01-06

**Authors:** Ishita Mostafa, Rahvia Alam Sthity, Umme Habiba Lamiya, Md. Tariqujjaman, Mustafa Mahfuz, S. M. Tafsir Hasan, Tahmeed Ahmed

**Affiliations:** 1 International Centre for Diarrhoeal Disease Research, Bangladesh (icddr,b), 68 Shaheed Tajuddin Ahmed Sarani, Mohakhali, Dhaka 1212, Bangladesh; rahvia.sthity@icddrb.org (R.A.S.); umme.lamiya@icddrb.org (U.H.L.); md.tariqujjaman@icddrb.org (M.T.); mustafa@icddrb.org (M.M.); tafsir.hasan@icddrb.org (S.M.T.H.); tahmeed@icddrb.org (T.A.); 2 Faculty of Medicine and Health Technology, Tampere University, 33100 Tampere, Finland; 3 Department of Public Health Nutrition, James P Grant School of Public Health, BRAC University, Dhaka 1212, Bangladesh

**Keywords:** microbiota-directed complementary food, moderate acute malnutrition, gut health, environmental enteric dysfunction, children, Bangladesh

## Abstract

Dietary supplementation with a gut microbiota-directed complementary food (MDCF-2) significantly improved weight gain and repaired gut microbiota, as reported in a recent randomized controlled trial on Bangladeshi children with moderate acute malnutrition (MAM). Environmental enteric dysfunction (EED) is a small bowel disorder, and recent evidence shows that it is linked to growth failure in children. Therefore, we intended to investigate whether supplementation with MDCF-2 has any role in modifying gut health by changing the levels of biomarkers of EED and gut inflammation in children with MAM. We randomly assigned 124 children aged 12–18 months to one of two intervention diets, either MDCF-2 or ready-to-use supplementary food (RUSF). Approximately 50 g of the diet was administered in two feeding sessions daily for 12 weeks. Stool and plasma biomarkers were assessed to evaluate intestinal health. Results showed that the average change in citrulline concentration (µmol/L) significantly increased among children who consumed MDCF-2 compared to those who consumed RUSF (mean difference-in-differences: 123.10; 95% CI: 3.60, 242.61; *p* = 0.044). The research findings demonstrated that MDCF-2 might have a beneficial effect on improving the gastrointestinal health of malnourished children.

## 1. Introduction

Acute malnutrition in early childhood is an alarming issue for low- and middle-income countries (LMICs) as cognitive, language, and motor development are impaired in malnourished children [1]. In 2022, 45 million children under 5 were affected worldwide with wasting. Among all the continents, Asia has borne the greatest share of malnutrition in the form of wasting (70%) [2]. Bangladesh is also suffering from this global health problem as 11% of under 5 children are wasted [3]. The prevalence of moderate acute malnourished (MAM) children is higher than that of children with severe acute malnutrition (SAM) and ~31 million under-5 children were moderately wasted among all those children who were acutely malnourished in 2022 [2]. The frequency of wasting might increase further due to global food insecurity, conflicts, and post-COVID-19 pandemic challenges [4,5]. Two criteria are assessed to define moderate acute malnutrition in children: (i) weight-for-length z-scores (WLZ) between <−2 and −3 compared to World Health Organization (WHO) Child Growth Standards and/or (ii) mid-upper-arm circumference (MUAC) greater or equal to 115 mm and less than 125 [6]. Although the main focus has been on the treatment of SAM children, children with MAM are also vulnerable, as their condition will progress to SAM if not treated properly. Efforts are being made to eradicate the burden, but, unfortunately, the progress toward achieving the global nutritional target by 2025 and the Sustainable Development Goal (SDG) by 2030 for childhood undernutrition is not satisfactory [2]. Considering the global context and complexity of intervention programs, a food-based nutrition intervention could be a solution to improve the nutritional status of under-5 children [7].

MAM increases the risk of death in children approximately threefold compared to healthy children [8]. The majority of the increase in childhood mortality attributable to malnutrition is due to an increased susceptibility to a diverse array of pathogens, particularly those causing gastrointestinal infections [9,10,11]. This increase in susceptibility to infection is most likely the result of intestinal injury, increased nutrient loss, malabsorption, and energy diversion to the immune response. These mechanisms resemble the effects of environmental enteric dysfunction (EED), which is a reversible, poorly characterized disorder of the small intestine [12]. EED is a common disorder of malnourished children living in poverty with repeated exposure to pathogens due to poor sanitary conditions and unhygienic environments in LMICs. Children living in resource-limited settings are exposed to multiple etiologies that make the nutritional impairments more complex [13]. Malnutrition-related intestinal changes are likely to be increased in children with EED [14,15]. According to UNICEF, insufficient dietary consumption is one of the most common causes of malnutrition [16]; along with this, the alteration in the gut microbiota plays a major role [17].

As the amount of food consumption is limited in malnourished children, an energy-dense food supplement is required to combat the battle with malnutrition. In order to determine the standard care for malnutrition, several food supplements have been introduced. However, these lipid-based, calorie-dense supplements could not sustain progress in growth and could not reduce relapse [18,19,20]. Most of the programs that aim to combat childhood undernutrition are based on a one-size-fits-all approach and do not take into account the biological differences that exist among individual children [21].

Recent evidence suggests that there is a relationship between EED and microbial dysbiosis in the gut as well as growth failure in children [22,23,24]. Therefore, a diet-based intervention targeting the gut microbiome can be useful to correct EED and associated gut microbial dysbiosis with a positive impact on growth and development during the early years of life [25]. Traditional therapeutic supplementation has already been proven to have minimal impact on gut microbiota repair; therefore, an alternative way to supplement malnourished children by targeting the immature gut microbiota was introduced by using locally available and culturally acceptable food ingredients [25,26]. Several recipes were tested and among these recipes, the lead microbiota-directed complementary food-2 (MDCF-2) proved to be one of a kind in repairing gut health [27]. MDCF-2 contains common ingredients that are easily available and inexpensive. When under-5 children with MAM were supplemented with this intervention, the gut microbiota of these children was repaired to an extent that resembled healthy children in the same community. The levels of plasma proteins related to neurodevelopment, immunity, and bone growth were also found to be positively associated with the supplementation of MDCF-2 [27].

In addition to restoring gut microbiota and being positively correlated with plasma proteins, MDCF-2 may potentially enhance the conditions of EED, an outcome that has not been previously investigated. In recent years, several studies have evaluated a range of potential non-invasive biomarkers to assess intestinal function. Myeloperoxidase (MPO) and Neopterin (Neo) are two such biomarkers that specifically indicate intestinal inflammation [28]. Alpha-1-antitrypsin (A1AT), a protease inhibitor, is a fecal biomarker used to assess protein-loss enteropathy [29]. Small bowel enterocytes generate citrulline, a plasma biomarker of EED, which indicates enterocyte mass as well as intestinal damage and repair [30,31]. Intestinal fatty acid-binding protein (I-FABP), only expressed by the intestinal mucosa, could be used to monitor intestinal mucosal damage and the risk of growth faltering in the future could also be assessed by this biomarker [32,33].

As MDCF-2 had a positive effect on repairing the gut microbiota of MAM children, we intended to investigate whether supplementation with MDCF-2 has any role in restoring gut health by changing the levels of biomarkers of gut health and EED in children with MAM.

## 2. Materials and Methods

### 2.1. Study Design

For this analysis, no patients were recruited; rather, data for this study were obtained from a randomized controlled trial, where dietary intervention was provided for 12 weeks to 124 children with MAM living in a slum settlement of the Mirpur area of Dhaka city, Bangladesh [34]. The study location of Mirpur was chosen due to its population consisting predominantly of impoverished and lower–middle-income households. The residential and sanitary conditions in Mirpur are representative of those commonly found in densely populated urban areas. The original study duration was 16 weeks, where intervention was provided for 12 weeks with 2 weeks of pre-intervention and 2 weeks of follow-up after the end of intervention. Anthropometric assessment was conducted periodically throughout the study and blood and stool samples were collected before and after the intervention and stored in the biorepository of icddr,b. After publishing the findings of the original trial elsewhere in 2021 [27], the rest of the stored biological samples, collected during that trial, were used to measure gut health and EED biomarkers.

### 2.2. Study Participants and Recruitment

Children aged 12 to 18 months with MAM and no bilateral pedal edema were eligible for enrollment. Children were screened for medical complications and underwent a comprehensive clinical evaluation to rule out other causes of secondary malnutrition. Children requiring hospital treatment for any medical condition, having tuberculosis (diagnosis based on WHO 2014 guidelines), any congenital/acquired disorder, a known case of trisomy-21 or cerebral palsy, persistent diarrhea, a known history of soy, peanut or milk protein allergy, severe anemia (8 mg/dL), and antibiotic use (within 15 days before initiation of intervention) were excluded from the study [34]. The study used a single-blind computer-generated randomization technique to recruit a total of 124 children, who were then allocated randomly to two distinct intervention arms, MDCF-2 and ready-to-use supplementary food (RUSF). Each arm consisted of an equal number of 62 children.

A group of qualified research assistants employed for this purpose carried out the participant screening and recruiting procedure. The parents were approached and provided with a comprehensive explanation of the study’s specifics. Subsequently, they were extended an invitation to enroll their children in the study, but only after the children met the predetermined eligibility criteria. This was accomplished by obtaining written informed consent from the parents of those individuals who were willing to take part.

### 2.3. Study Interventions

Samples were prepared in the icddr,b specialized kitchen, which follows standardized production procedures to control the quality of MDCF from each production batch and ensure that no unexpected contamination or nutrient losses occur during processing. MDCF-2 is composed of locally sourced components, including chickpea, soybean, peanut, green banana, sugar, and soybean oil and added with vitamin–mineral premix. The composition of RUSF includes rice, lentils, milk powder, sugar, soybean oil, and a vitamin–mineral premix. The participants were given these diets as a supplement to their regular diet. The RUSF supplement offered around 250 Kcal of energy, while the MDCF-2 supplement gave approximately 215 Kcal of energy.

### 2.4. Sample Size Calculation

To estimate sample size, we considered a baseline WLZ of -2 and an endline WLZ of −1.7 with a pooled standard deviation of 0.53 from a pre-POC MDCF study. We calculated that a sample size of 62 was required for each arm to test whether MDCF-2 exhibits superior efficacy (ponderal growth, host biomarkers of a biological state) than a conventional RUSF diet in children with MAM over an intervention period of three months, considering 80% power, a 5% level of significance, and 20% attrition. Therefore, a total of 124 children were included in both arms. A detailed description of sample size has been published elsewhere [34].

### 2.5. Data Collection

At baseline, data relevant to the socio-demographic attributes (maternal age and completed years of education, family’s assets, income, and access to improved toilet facilities, etc.) and anthropometric data were obtained. We also obtained data on water, sanitation, and hygiene practices. During the intervention, morbidity data related to common illnesses such as fever, diarrhea, stomach pain, nausea, vomiting, abrasion, skin itching, toothache, constant cough, runny nose, and panting were collected daily.

### 2.6. Biological Sample Collection and Analysis

Two mL of venous blood and 6 g of fecal samples were obtained before and after the intervention period. Plasma was separated by centrifuging at 2500 rpm for 10 min from each blood sample and all the plasma as well as the stool samples were stored until analysis at a −80 °C freezer in the biorepository of icddr,b. Using enzyme-linked immunosorbent assay (ELISA) kits (commercially available), citrulline (Abcam, Waltham, MA, USA) and I-FABP (R&D Systems, Minneapolis, MN, USA) were measured from plasma and MPO (Immundiagnostik, Bensheim, Germany), NEO (GenWay Biotech, San Diego, CA, USA), and A1AT (Biovendor, Chandler, NC, USA) were measured from fecal samples. The laboratory assays were performed at the Emerging Infections & Parasitology Laboratory of icddr,b.

### 2.7. Statistical Analysis

The sample characteristics of both children and their mothers were presented in percentages with numbers for categorical variables. For continuous variables, we presented the mean with standard deviation (SD). We examined the values of the plasma biomarkers, citrulline and I-FABP, and fecal biomarkers, A1AT, MPO, and NEO, before the start of the intervention and at the end of the 3-month intervention. We checked the distribution of the biomarkers visually using a histogram with a superimposed normal curve and quantile–quantile (Q–Q) plot. Citrulline was normally distributed. We log-transformed I-FABP, A1AT, MPO, and NEO before all analyses, as these variables were log-normally distributed. We summarized the biomarker data using mean and SD. We performed unadjusted and multivariable difference-in-differences (DID) analysis using a linear regression platform to explore the effect of MDCF-2 on changes in the levels of the biomarkers mentioned above. The multivariable model was adjusted for child sex, WLZ, and LAZ. DID was expected to provide a more robust effect estimate because there was a difference in the levels of biomarkers between the groups at the baseline, and biomarkers were not the primary endpoints in the original randomized controlled trial, nor did the sample size estimation consider changes in these endpoints. The estimates were presented in coefficients with a respective 95% confidence interval (CI). The *p*-value of <0.05 was considered statistically significant. We performed a complete case analysis and dropped observations with biomarkers having any missing values. Statistical software package Stata, version 15.0 SE (Stata Corp. LP, College Station, TX, USA), was used to analyze the data.

## 3. Results

**Figure 1:**
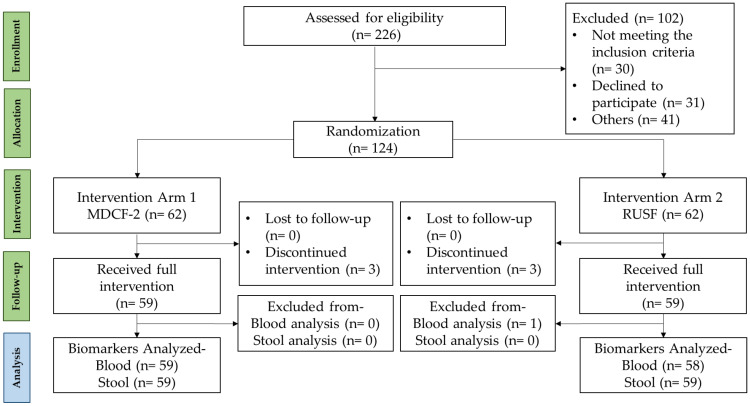
Study flow diagram.

As depicted in Figure 1, a comprehensive assessment was conducted on a total of 226 children to determine their eligibility. One hundred twenty-four children were enrolled in the trial since they met the eligibility criteria, while the remaining children refused to participate or did not satisfy the eligibility criteria. Out of 124 participants, 118 (95.16%) completed the 16-week follow-up period. At the beginning and at the end of the 12-week intervention period, 117 (MDCF-2: 59, RUSF: 58) of these participants provided blood samples and stool specimens were collected from 118 participants (MDCF-2: 59, RUSF: 59).

The socio-demographic characteristics of participants are shown in Table 1. The average age of children in the MDCF-2 group was 15.31 (±1.93 SD) months, slightly lower than the average age of 15.51 (±2.01 SD) months in the RUSF group. The distribution of sex in both groups was nearly identical, with 44% male children in the MDCF-2 group and 42% male children in the RUSF group. Interestingly, we observed a higher proportion of children suffering from illness in the MDCF-2 group (60%) compared to the RUSF group (37%). The average weight-for-length z-score in the MDCF-2 group was −2.31, while in the RUSF group, it was −2.40. Other characteristics of children and mothers, including child’s height, child’s weight, child’s MUAC, mother’s age, and the number of household members, followed a similar pattern in both groups, as detailed in Table 1.

According to the findings shown in Table 2, it was observed that the average stool MPO concentration in the baseline was 7.67 in the MDCF-2 group, slightly lower than the corresponding value of 7.79 in the RUSF group. Similarly, a lower concentration of NEO (6.81) was observed in the MDCF-2 group compared to the RUSF group (6.96). On the contrary, the average A1AT concentration was higher in the MDCF-2 group (−0.41) compared to the RUSF group (−0.50). Turning to plasma biomarkers, in the MDCF-2 group, the average concentration of citrulline was lower (517.44) than in the RUSF group (661.82). I-FABP exhibited higher concentrations in the MDCF-2 group.

According to the data presented in Table 3, the results of the difference-in-differences analysis examine the effect of MDCF-2 on plasma and stool biomarkers. The average change in citrulline concentration significantly increased among children who consumed MDCF-2 compared to those who consumed RUSF (coefficient: 123.10; 95% CI: 3.60, 242.61; *p* = 0.044). However, the effects of MDCF-2 on the other plasma biomarkers were in the opposite direction, although these differences did not reach statistical significance. Regarding stool biomarkers, the effect of MDCF-2 on A1AT was negative and statistically significant at the 25% level (coefficient: −0.28; 95% CI: −0.73, 0.17; *p* = 0.221). The effects of MDCF-2 on the other two stool biomarkers were not statistically significant.

## 4. Discussion

This study employed a two-arm, randomized controlled trial designed to investigate the effect of the MDCF-2 on Bangladeshi children with MAM, compared to RUSF. Following a three-month experiment involving the daily administration of MDCF and RUSF to children with MAM, there was a statistically significant rise in citrulline, a biomarker of gut health. However, not all of the tested biomarkers showed the same level of significance.

A systematic review by Harper et al. examined the role of intestinal damage and repair as one of the five domains in elucidating the intricate process via which EED might be implicated in stunting. Citrulline and I-FABP are both used to explore the potential correlations between noninvasive indicators of gastrointestinal damage and repair with stunting [23]. The levels of citrulline in circulation are an indicator of the total mass of enterocytes, and low levels suggest a decrease in the surface area [35]. We have quantified both of these biomarkers and our findings revealed a statistically significant elevation in citrulline concentration among children in the group supplemented with MDCF-2 as compared to those in the RUSF group. Consequently, any rise in this biomarker could potentially be interpreted as an indicator of a decline in intestinal damage and an increase in enterocyte mass, which would be a favorable consequence. Other investigators have also observed similar findings, indicating that implementing a gluten-free diet intervention leads to a considerable rise in citrulline levels among children diagnosed with Coeliac disease [36]. A study conducted in rural Malawi revealed that increased intestinal permeability was significantly associated with lower serum citrulline along with other metabolites in children aged 12–59 months. This association between low levels of circulating citrulline and increased permeability in the intestines may indicate a decrease in the overall number of enterocytes in the small intestine [37]. I-FABP is released quickly into the bloodstream following damage to the epithelial cells and has a brief half-life, which indicates recent damage to the gut. It has been employed to denote the extent of gastrointestinal harm in children [38]. The results of our study did not show any significant decline in I-FABP levels in children who received MDCF-2 supplementation, compared to the children who participated in the RUSF group. This result aligns with previous findings from a study conducted in the Netherlands, indicating that persons with Coeliac disease did not have a notable decrease in the amount of I-FABP after supplementing with a gluten-free diet for up to 3 years [39].

The establishment of cut-off values for the common EED biomarkers (MPO, A1AT, and NEO), which play a role in intestinal inflammation, in children with malnutrition has not yet been accomplished [40]. In the present investigation, we assessed the levels of MPO, A1AT, and NEO before and following the intervention and we did not find any statistical significance between the intervention groups. The MDCF-2 exhibited a drop in A1AT, which would be statistically significant at the 20% level. Due to the limited duration of the interventions and the relatively small sample size, the observed decrease in the biomarker level probably lacks statistical significance. Previous research conducted on children has yielded varying results. For instance, a study was undertaken in Nepal to explore the relationships between nutrient consumption (potassium, magnesium, phosphorous, folate, and vitamin C) and indicators of EED. The findings demonstrated that the intake of nutrients played a role in decreasing intestinal inflammation by reducing the level of MPO [41]. However, a separate study carried out in Bangladesh determined that providing yogurt as a dietary intervention to children aged 5–6 months, who were at risk of stunting, for three months did not result in significant effects on fecal biomarkers [42].

As the initial trial aimed to determine the efficacy of MDCF-2 in enhancing the development of gut microbiota that promote growth, our main focus was to analyze changes in gut health and EED biomarkers. In our earlier study, we used linear mixed-effects models to figure out if WLZ is positively related to the proportion of growth-promoting microbiota in the gut and to measure the effects of supplementation on the configuration of the microbial community. The study identified 21 bacterial taxa that were positively correlated with a higher WLZ score in the MDCF-2 group compared to the RUSF group [27].

The MDCF-2 for malnourished children is crucial to public health. It is designed to meet the acute health needs of undernourished children and is more effective than traditional complementary foods. In addition, they can restore gut health, are enriched with essential nutrients, and are crucial for the healthy growth and development of children [25]. Their adaptability for use in various geographical regions underscores their significance in the global fight against malnutrition.

To ensure the program’s success in eliminating malnutrition worldwide, this intervention can be manufactured locally at a reasonable cost while upholding safety standards and considering the age and nutritional requirements of the children. Sustainability is imperative for the implementation of such initiatives.

### Strengths and Limitations

The strengths of the study include the single-blinded randomization technique to enroll study participants and directly observed feeding monitored by trained field staff. Using advanced laboratory techniques to quantify the plasma and stool biomarkers is another notable strength of the study. Nevertheless, we acknowledge a few limitations. First, due to the nature of the intervention, we could not ensure double-masking. Second, while biopsy studies examining the histological features of the intestinal mucosa are currently being conducted in Bangladesh [43], we did not collect intestinal biopsy samples from the children in our study. Finally, this study was exploratory in nature and the sample size determination did not consider changes in the fecal and plasma biomarkers evaluated in this study.

## 5. Conclusions

In conclusion, the concentration of citrulline is higher in the children following 12 weeks of MDCF-2 supplementation compared to those in a RUSF supplementation group. Elevated levels of citrulline suggest that there is an improvement in intestinal repair and an increase in the mass of enterocytes in children who received MDCF-2. Nevertheless, it is necessary to conduct thorough biomarker screening and subsequent research to identify more potential candidates for biomarkers of gut health and EED.

## Figures and Tables

**Table 1:** Baseline characteristics of the participating children, by study group.

Variables	MDCF (*n* = 62)	RUSF (*n* = 62)
Child age (month) ^1^	15.31 (±1.93)	15.51 (±2.01)
Males ^2^	43.55 (27)	41.94 (26)
Weight (kg) ^1^	7.32 (±0.67)	7.28 (±0.64)
Length (cm) ^1^	72.97 (±3.53)	73.06 (±3.17)
MUAC (in cm) ^1^	12.78 (±0.53)	12.69 (±0.44)
Any illness symptoms in the preceding 7 days ^2,3^	59.68 (37)	37.10 (23)
Weight-for-length z-score ^1^	−2.31 (±0.29)	−2.40 (±0.27)
Length-for-age z-score ^1^	−2.08 (±1.15)	−2.08 (±1.12)
Maternal age (years) ^1^	23.92 (±5.05)	24.25 (±4.75)
Number of family members ^1^	5.03 (±2.89)	4.94 (±1.75)

Values represent: ^1^ mean (±SD); ^2^ number (percentage); ^3^ common illnesses include fever, diarrhea, stomach pain, nausea, vomiting, abrasion, skin itching, toothache, constant cough, runny nose, panting. Abbreviations: SD, standard deviation; MUAC, mid-upper-arm circumference; Length, recumbent length.

**Table 2:** Baseline comparison of biomarkers between two groups.

Biomarkers	MDCF-2 Group, Mean (±SD)	RUSF Group, Mean (±SD)
Stool MPO (ng/mL) in log scale	7.67 (±0.99)	7.79 (±1.18)
Stool NEO (nmol/L) in log scale	6.81 (±1.09)	6.96 (±1.12)
Stool A1AT (mg/mL) in log scale	−0.41 (±0.90)	−0.50 (±0.82)
Plasma Citrulline (µmol/L)	517.44 (±211.45)	661.82 (±233.82)
Plasma I-FABP (ng/mL) in log scale	0.70 (±0.47)	0.65 (±0.47)

Abbreviations: MPO, Myeloperoxidase; NEO, Neopterin; A1AT, alpha-1-antitrypsin; I-FABP, intestinal fatty acid-binding protein; SD, standard deviation.

**Table 3:** Difference in difference analysis of the effect of MDCF-2 on plasma and stool biomarkers.

Biomarkers	Mean Diff-in-Diff ^1^ [95% CI]	*p*-Value	Mean Diff-in-Diff ^2^ [95% CI]	*p*-Value
Plasma				
Plasma Citrulline (µmol/L)	126.78 [3.59, 242.60]	0.036	123.10 [3.60, 242.61]	0.044
Plasma I-FABP (ng/mL) in log scale	−0.098 [−0.42, 0.17]	0.517	−0.13 [−0.42, 0.17]	0.404
Stool				
Stool MPO (ng/mL) in log scale	−0.11 [−0.65, 0.43]	0.687	−0.11 [−0.65, 0.43]	0.693
Stool NEO (nmol/L) in log scale	−0.078 [−0.68, 0.52]	0.798	−0.068 [−0.66, 0.53]	0.821
Stool A1AT (mg/mL) in log scale	−0.31 [−0.77, 0.14]	0.173	−0.28 [−0.73, 0.17]	0.221

^1^ Difference-in-differences analysis using a simple linear regression platform; ^2^ difference-in-differences analysis using multiple linear regression adjusted for child sex, length-for-age z-score, and weight-for-length z-score. Abbreviations: MPO, Myeloperoxidase; NEO, Neopterin; A1AT, alpha-1-antitrypsin; I-FABP, intestinal fatty acid-binding protein; CI, confidence interval.

## Data Availability

The data presented in this study are available on request from the corresponding author. The data are not publicly available due to privacy issues.
